# 2704. Real-world safety and effectiveness of letermovir for preventing cytomegalovirus (CMV) disease in patients undergoing allogenic hematopoietic stem cell transplantation (allo-HSCT): Final results of post-marketing surveillance (PMS) in Japan

**DOI:** 10.1093/ofid/ofad500.2315

**Published:** 2023-11-27

**Authors:** Masaki Fukuda, Junko Hattori, Rika Ohkubo, Asuka Watanabe, Shinichiroh Maekawa

**Affiliations:** MSD, KK, Chiyoda-ku, Tokyo, Japan; MSD K.K., Tokyo, Tokyo, Japan; MSD K.K., Tokyo, Tokyo, Japan; MSD K.K, Tokyo, Tokyo, Japan; MSD K.K, Tokyo, Tokyo, Japan

## Abstract

**Background:**

CMV is one of the most common opportunistic infections in patients undergoing allo-HSCT. Letermovir is an inhibitor of CMV DNA terminase that is approved for CMV prophylaxis in patients undergoing allo-HSCT. We report the final results of a PMS that collected data on its safety and effectiveness of letermovir in real-world clinical practice after its approval in Japan.

**Methods:**

The case report forms were drafted in part by the Japanese Data Center for Hematopoietic Cell Transplantation using data elements in the Transplant Registry Unified Management Program and sent to individual HSCT centers to decrease the burden of reporting. Patients who underwent HSCT and started letermovir between May 2018–May 2022 at one of 136 centers in Japan were registered in the PMS. Safety data collected in the PMS included adverse events (AEs)/adverse drug reactions (ADRs), which were assessed by the physicians. Clinical effectiveness outcomes were the development of CMV disease, CMV antigen status, and use of preemptive therapy within 48 weeks of starting letermovir. The database was locked on October 5, 2022.

**Results:**

Overall, 932 patients who underwent HSCT were registered, of which 821 were included in safety analyses. ADRs occurred in 11.33% of patients and serious ADRs occurred in 3.05%. The five most common ADRs were nausea (1.58%), renal impairment (1.46%) and acute graft versus host disease (0.61%), CMV test positive (0.61%), and hepatic function abnormal (0.61%). AEs related to renal disorders occurred in 6.67% and 4.30% of patients treated with intravenous and oral letermovir, respectively. AEs related to cardiac disorders occurred in 3.05% of patients treated with oral/intravenous letermovir. Of 670 patients eligible for the effectiveness analyses at Week 48, CMV antigens were detected in 38.36% (Figure 1).

**Figure:**
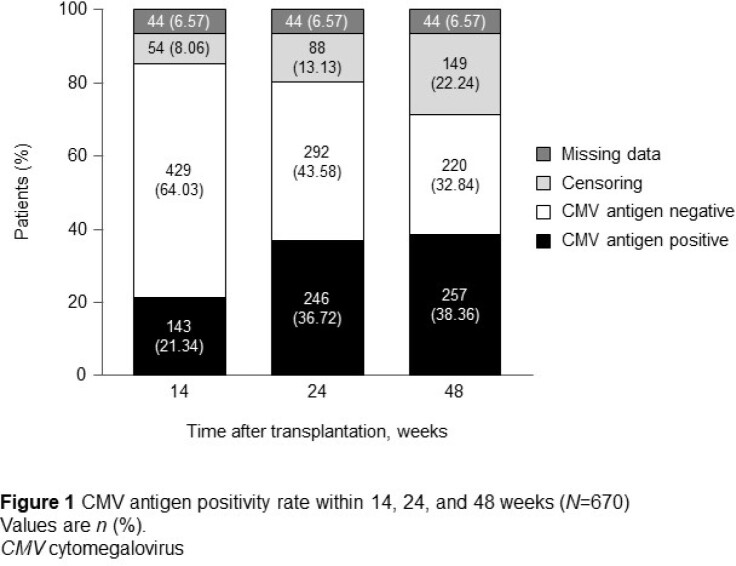

**Conclusion:**

This final analysis PMS in Japan provides further evidence supporting the effectiveness and long-term safety of letermovir for CMV prophylaxis in patients undergoing allo-HSCT in real-world settings.

**Disclosures:**

**Masaki Fukuda**, MSD K.K.: Grant/Research Support|MSD K.K.: Salary provided by MSD K.K. **Junko Hattori, PhD**, MSD, KK: Grant/Research Support|MSD, KK: Salary **Rika Ohkubo, PhD**, MSD K.K.: Grant/Research Support|MSD K.K.: Salary provided by MSD K.K. **Asuka Watanabe, n/a**, MSD K.K.: Grant/Research Support|MSD K.K.: Salary provided by MSD K.K. **Shinichiroh Maekawa, n/a**, MSD K.K.: Grant/Research Support|MSD K.K.: Salary provided by MSD K.K.

